# Transcriptomic Analysis of Long Noncoding RNA and mRNA Expression Profiles in the Amygdala of Rats with Bone Cancer Pain-Depression Comorbidity

**DOI:** 10.3390/life11080834

**Published:** 2021-08-14

**Authors:** Shuyan Wu, Xiaohui Chen, Fengyi Huang, Mingxue Lin, Pinzhong Chen, Haiyang Wan, Fei Gao, Ting Zheng, Xiaochun Zheng

**Affiliations:** 1 Department of Anesthesiology, Shengli Clinical Medical College of Fujian Medical University, Fuzhou 350001, China; 2191003810@fjmu.edu.cn (S.W.); chenxh@fjmu.edu.cn (X.C.); huangfy@fjmu.edu.cn (F.H.); ccdjzzi@fjmu.edu.cn (M.L.); chenpinzhong@fjmu.edu.cn (P.C.); 2191003809@fjmu.edu.cn (H.W.); gaofei@fjmu.edu.cn (F.G.); zhengting@fjsl.com.cn (T.Z.); 2 Department of Anesthesiology, Fujian Provincial Hospital, Fuzhou 350001, China; 3 Fujian Provincial Institute of Emergency Medicine, Fujian Provincial Key Laboratory of Emergency Medicine, Fuzhou 350001, China

**Keywords:** bone cancer pain, depression, high-throughput RNA sequencing, inflammation, immune response

## Abstract

Bone cancer pain (BCP)–depression comorbidity has become a complex clinical problem during cancer treatment; however, its underlying molecular mechanisms have not been clarified. Several long noncoding RNAs (lncRNAs) have been demonstrated to be promising therapeutic targets in depression, but research on the role of lncRNAs in BCP–depression comorbidity has been limited. Therefore, high-throughput RNA sequencing was performed to detect differentially expressed profiles in the amygdala of a BCP–depression rat model in this study. We detected 330 differentially expressed mRNAs (DEmRNAs) and 78 differentially expressed lncRNAs (DElncRNAs) in the BCP–depression comorbidity model and then verified the expression of six DEmRNAs and six DElncRNAs with the greatest degrees of difference by RT-qPCR. Furthermore, Gene Ontology (GO) and Kyoto Encyclopedia of Genes and Genomes (KEGG) analyses revealed that differentially expressed genes were strongly enriched in inflammatory and immunologic systemic responses. Then the nuclear factor kappa B (NF-κB) signaling pathway and the Th17 differentiation pathway showed significant differences, as determined by Western blot analysis. Finally, we constructed a protein–protein interaction (PPI) network to explore the potential regulatory mechanism of DEmRNAs. In conclusion, our study reveals a new resource for the understanding of dysregulated lncRNAs and mRNAs in BCP–depression comorbidity and provides novel potential therapeutic targets for further approaches.

## 1. Introduction

Primary and metastatic cancers that invade the bone are frequently associated with severe and intractable bone cancer pain (BCP) [1]. BCP can lead to depression and seriously affect the living quality of patients; the severity of the depression, in turn, also greatly affects the occurrence of BCP by aggravating the patient’s perception of pain and reducing the effect of analgesic treatment [2,3,4]. According to the latest data, 19.29 million new cases of cancer occur each year worldwide [5], and bone metastasis is the most common type of tumor metastasis in patients with advanced cancer [6], which increases the number of patients who suffer from BCP–depression comorbidity. However, the pathological mechanism of BCP–depression comorbidity has not been well-studied yet.

Long noncoding RNAs (lncRNAs) are highly conserved RNAs of more than 200 nucleotides in length [7]. Gene regulation, especially lncRNA regulation at the transcription level, is a critical part of gene expression modulation in all living organisms [8]. Studies have supported that lncRNAs may contribute to the molecular etiopathogenesis of BCP or depression by regulating gene expression [9,10,11,12,13], but they remain poorly understood in the BCP–depression comorbidity. Furthermore, the amygdala is a brain region associated with both pain and depression [14,15,16,17], and the role of the amygdala in the BCP–depression comorbidity has not been reported. Therefore, it is much more crucial to elucidate specific lncRNAs and their underlying molecular mechanism of the BCP–depression comorbidity, which in turn will provide information on which potential treatments can be designed.

To evaluate current therapies and develop new medical treatments, we performed transcriptome sequencing of the contralateral amygdala of rats with BCP–depression comorbidity to investigate differentially expressed profiles of lncRNAs and mRNAs and attempted to provide new information for the comorbidity of BCP and depression through the functional analysis of differentially expressed genes (DEGs).

## 2. Materials and Methods

### 2.1. Experimental Animals

Female Sprague Dawley rats (150–180 g) were provided by the Experimental Animal Center of Fujian Medical University and group-housed in cages at 22 ± 2 °C with a 12 h/12 h alternating light/dark cycle and free access to food and water. Our experimental methods were approved by the Experimental Animal Care and Use Committee of Fujian Medical University (Fujian, China) and complied with the guidelines for pain research on laboratory animals [18].

### 2.2. Surgical Procedure to Establish a Rat Model of BCP

As previously described [19,20], MRMT-1 carcinoma cells were implanted into the tibial marrow cavity to establish the rat model of BCP, and on postoperative day 14, malignant ascites were collected, rinsed, centrifuged aseptically, washed three times with D-Hank’s solution (Boster, Wuhan, China), and diluted to 2 × 10^7^ cells/mL for inoculation. Animals were intraperitoneally injected with 2% phenobarbital sodium (40 mg/kg) for deep anesthesia. The right hind paw of the rats was sterilized three times with a 7% tincture of iodine and shaved above the tibial plateau using a hair clipper, after which an incision was performed to expose the tibia. Next, the right tibial plateau was drilled with a 50-μL microsyringe, and the marrow cavity was slowly injected with 10 μL of MRMT-1 carcinoma cell suspension (or 10 μL of D-Hank’s solution without cells in sham rats) through the hole. Bone wax was used to seal the needle hole, and the incision was interruptedly sutured after washing with 75% ethanol.

Tibial X-ray and hematoxylin and eosin (H&E) staining were performed 14 days after BCP modeling to determine bone destruction and evaluate tumor growth.

### 2.3. Behavioral Tests

Mechanical withdrawal threshold (MWT): The MWT was assessed by measuring the paw withdrawal mechanical threshold (PWMT) using von Frey filaments at day 0 before the operation and on days 7, 14, and 21 after the surgery. Each rat was placed in a transparent box (26 cm × 12 cm × 12 cm) and habituated for 30 min before the test; then, the MWT was measured with von Frey filaments ranging in force from 0.4 g to 26 g (0.4, 0.6, 1, 1.4, 2, 4, 6, 8, 10, 15, and 26 g) stabbing perpendicularly to the hind paw, following the methods a previous study [21,22]. A positive response was defined as quick paw withdrawal, shaking, biting, or licking. Data were analyzed by averaging three values after measuring the MWT three times for each rat.

Sucrose preference test (SPT): Rats were adapted to drinking sucrose water before the operation, and the baseline sucrose preference was then assessed. After fasting for 24 h, rats were given a preweighed bottle of 1% sucrose water and drinking water within 1 h. The position of the bottle was changed every 30 min. Finally, the liquid consumed from both bottles was determined by weighing the remaining liquid. Sucrose preference (%) was calculated as sucrose water consumption divided by total liquid consumption. Rats with a preoperative SPT index greater than or equal to 65% were included.

Forced swimming test (FST): Rats were separated into a cylindrical container (20 cm in diameter and 50 cm in height) filled with water (24–26 °C) and subjected to adaptive swimming for 15 min during the pretest. After 24 h, the rats were put back into the water tank. The rats were adapted to swimming for 1 min, and the immobility time within 5 min (the total time of rats floating in the water without struggling) was recorded by video. Two researchers blinded to the group assignment were trained to keep track of the resting time by a manual stopwatch. The results are the averages of the two researchers’ measurements. The water in the tank was changed after each rat. The test took place between 8 a.m. and 4 p.m.

### 2.4. Tissue Collection, RNA Extraction, Library Construction, and Sequencing

On the 28th day after surgery, all rats were thoroughly anesthetized and sacrificed mechanically by decapitation. The contralateral amygdala was quickly isolated, and meninges were removed; then, the fresh samples were snap-frozen in liquid nitrogen and preserved at −80 °C.

Total RNA was extracted utilizing a TRIzol reagent kit (Invitrogen, Carlsbad, CA, USA) in accordance with the manufacturer’s protocol. The quality of the RNA was evaluated on an Agilent 2100 Bioanalyzer (Agilent Technologies, Santa Clara, CA, USA) and verified using RNase-free agarose gel electrophoresis. Following the extraction of total RNA, eukaryotic mRNA was enriched with oligo(dT) beads and fragmented by fragmentation buffer. Next, the enriched mRNA was reverse transcribed into cDNA with randomized primers. Second-strand cDNA was synthesized using DNA polymerase I, RNase H, dNTPs, and buffer. Subsequently, the cDNA fragments were purified using a QiaQuick PCR extraction kit (Qiagen, Limburg, the Netherlands) and end-repaired; poly(A) was added, and the fragments were ligated to illumina sequencing adapters. PCR amplification products were selected by agarose gel electrophoresis and sequenced using an Illumina NovaSeq6000 from Gene Denovo Biotechnology Co. (Guangzhou, Guangdong, China).

### 2.5. Quantitative Reverse Transcription PCR (RT-qPCR)

The sequence data were validated using a real-time quantitative PCR system (StepOnePlus, Applied Biosystems, Foster City, CA, USA). Briefly, total RNA was extracted as described above and reverse-transcribed according to the manufacturer’s instructions (Vazyme Biotech Co., Ltd., Nanjing, China). The primer sequences are listed in Supplementary Table S1.

### 2.6. Western Blot Analysis

After sacrificing the rats of both groups, the amygdalae were removed quickly. The tissue was immediately frozen and lysed in RIPA buffer containing protease and phosphatase inhibitors (Beyotime Institute of Biotechnology, Shanghai, China). The concentration of protein lysates was determined by a BCA protein assay kit (Beyotime). The proteins were transferred to polyvinylidene fluoride membranes after separation by electrophoresis (Millipore, Munster, Ireland) and were then incubated with primary antibodies after blocking with 5% nonfat dry milk in TBST. The primary antibodies included rabbit anti-TGF-β (1:1000, ab215715, Abcam), rabbit anti-IL-6 (1:1000, ab233706, Abcam), rabbit anti-NF-κB p105/p50 (1:1000, ab32360, Abcam), rabbit anti-NF-κB p105/p50 (phospho S337) (1:1000, ab194729, Abcam), anti-IκBα (1:1000, ab194729, Abcam), and anti-IκBα (phospho S36) (1:1000, ab194729, Abcam), which were used according to standard procedures. The blots were visualized by ECL (Thermo Fisher Scientific, Invitrogen, Carlsbad, CA, USA), and quantification analysis was performed (Bio-Rad Laboratories, Hercules, CA, USA).

### 2.7. Targets Prediction of DElncRNAs

By comparing transcripts in known protein databases, two software programs, CNCI Utilizing Sequence Intrinsic Composition to Classify Protein-Coding and Long Non-Coding Transcripts [23] (version 2) and CPC [24] (version 0.9-r2), were used to predict the protein-coding potential of novel transcripts using default parameters. The intersection of the two prediction results of non-protein-coding transcripts was selected as lncRNA.

### 2.8. Gene Ontology (GO) Annotation and Kyoto Encyclopedia of Genes and Genomes (KEGG) Pathway Enrichment Analysis

GO has three ontologies: molecular function (MF), cellular component (CC), and biological process (BP). First, all DEGs were mapped to GO terms within the GO database (http://www.geneontology.org/ in 1 June 2021), the numbers of genes were computed for each term, and the GO terms significantly enriched in the DEM compared with the genome background were determined by a hypergeometric assay. The calculated *p*-value was corrected by the FDR, assuming FDR = 0.05 as the threshold, and the top 25 terms (based on the *p*-value) of each class were displayed above the bar graph. KEGG is the main publicly available database of pathways [25]. KEGG analysis was used to identify significantly enriched metabolic pathways and signal transduction pathways among the DEGs, and the top 20 pathways were shown in a bubble map. The calculation formula was the same as that in the GO analysis.

### 2.9. Construction of a Protein–Protein Interaction (PPI) Network Using Data of the Top 200 Differentially Expressed mRNAs (DEmRNAs)

Using String v10 [26], proteins encoded by the top 200 DEmRNAs were selected to construct a PPI network, in which genes were represented as nodes and interactions as lines.

### 2.10. Statistical Analysis

All behavioral and biochemical data are shown as means ± standard deviation (SD). For the MWT data, a two-way ANOVA with repeated measures was used to detect differences between the two groups, followed by Bonferroni post hoc analysis for multiple comparisons. For SPT, FST, and all biochemical data, a Student’s t-test was used to compare the differences between groups. A value of *p* < 0.05 was considered statistically significant.

## 3. Results

### 3.1. Implantation of MRMT-1 Carcinoma Cells Led to the Development of Mechanical Allodynia, Osteolytic Lesions, and Depressive Behaviors

**Figure 1:**
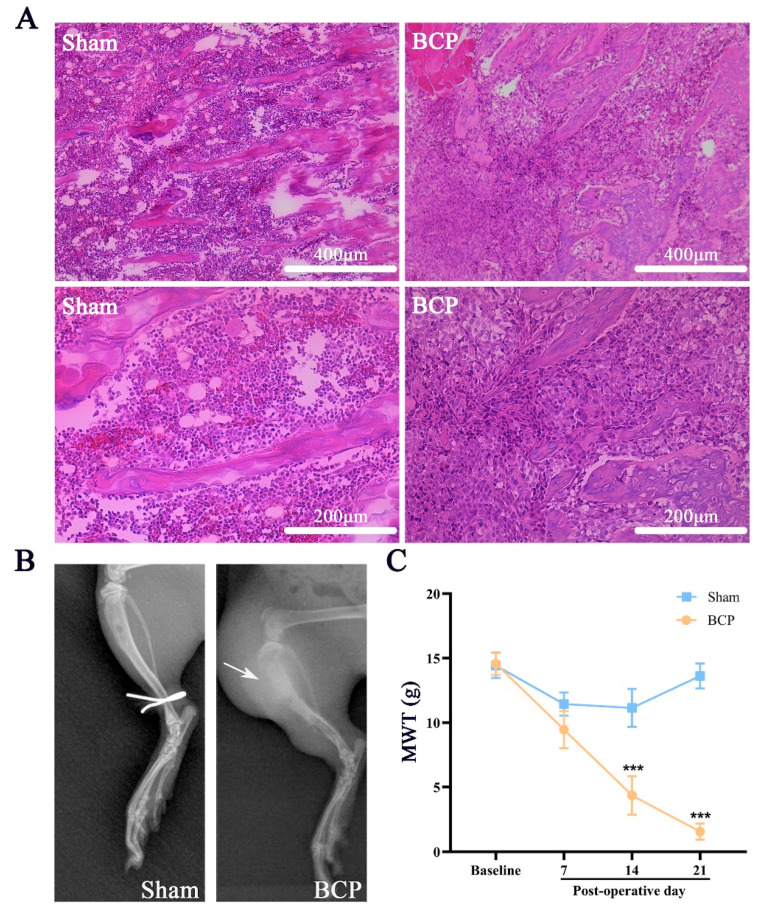
Bone destruction and hyperalgesia induced by intratibial implantation of MRMT-1 carcinoma cells. (**A**) The tibial trabeculae of BCP rats were destroyed and disarranged, as displayed by microscopic examination of tibial slices with hematoxylin and eosin (HE) staining. Scale bar = 400 μm (upper plots) and 200 μm (bottom plots). (**B**) Reduction in bone mineral density and discontinuity of cortical bone (marked by the white arrow) shown by X-rays of right hind paws indicated that the carcinoma cells induced bone destruction. (**C**) The MWT showed a significant downward tendency in BCP rats compared with sham rats from 7 days after modeling and was maintained until the end of the behavioral test. The data are expressed as means ± SD (*n* = 7 per group). *** *p* < 0.001 in comparison with sham rats at each timepoint; BCP, bone cancer pain; MWT, mechanical withdrawal threshold.

As shown in Figure 1, we found that cancer-bearing rats displayed severe destruction of cortical bone and disarrangement of bone trabecula of the tibia bone compared with sham rats, which was demonstrated by pathological and radiological examination, indicating osteolytic lesions after modeling (Figure 1A,B). Additionally, increased responsiveness to mechanical stimuli of the hind paw on postoperative days 7, 14, and 21 was observed in cancer-bearing rats compared with sham rats, in which responsiveness remained unchanged (Figure 1C, *p* < 0.001).

**Figure 2:**
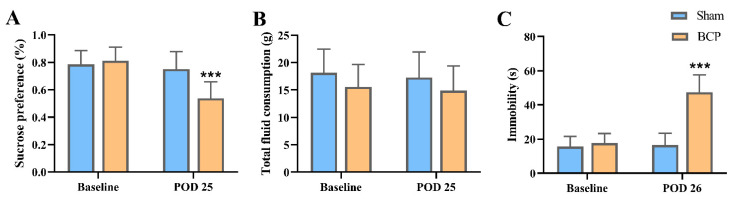
BCP rats developed depressive-like behaviors on POD 25 and 26. (**A**) Sucrose preference decreased in BCP rats as shown by the SPT on POD 25 compared with that in sham-operated rats. (**B**) The total intake volume in the BCP–depression and sham groups. (**C**) BCP rats showed extended immobility time in the FST on POD26. The data are expressed as means ± SD (*n* = 7 per group). *** *p* < 0.001 compared with sham rats at each timepoint. SPT, sucrose preference test; FST, forced swimming test; BCP, bone cancer pain group, POD, postoperative day.

Rats that underwent the SPT and FST before surgery were subjected to the SPT and FST again 25–26 days after surgery, and the results of the SPT showed a decreased preference for sucrose on postoperative day (POD) 25 (Figure 2A), while the total fluid consumption was not altered in either group (Figure 2B). The immobility time of rats with BCP increased significantly on POD 26 (Figure 2C). Therefore, BCP induced decreased sucrose preference and an increase in immobility in the FST.

### 3.2. Changes in Gene Expression Profiles Detected by High-Throughput RNA Sequencing

**Figure 3:**
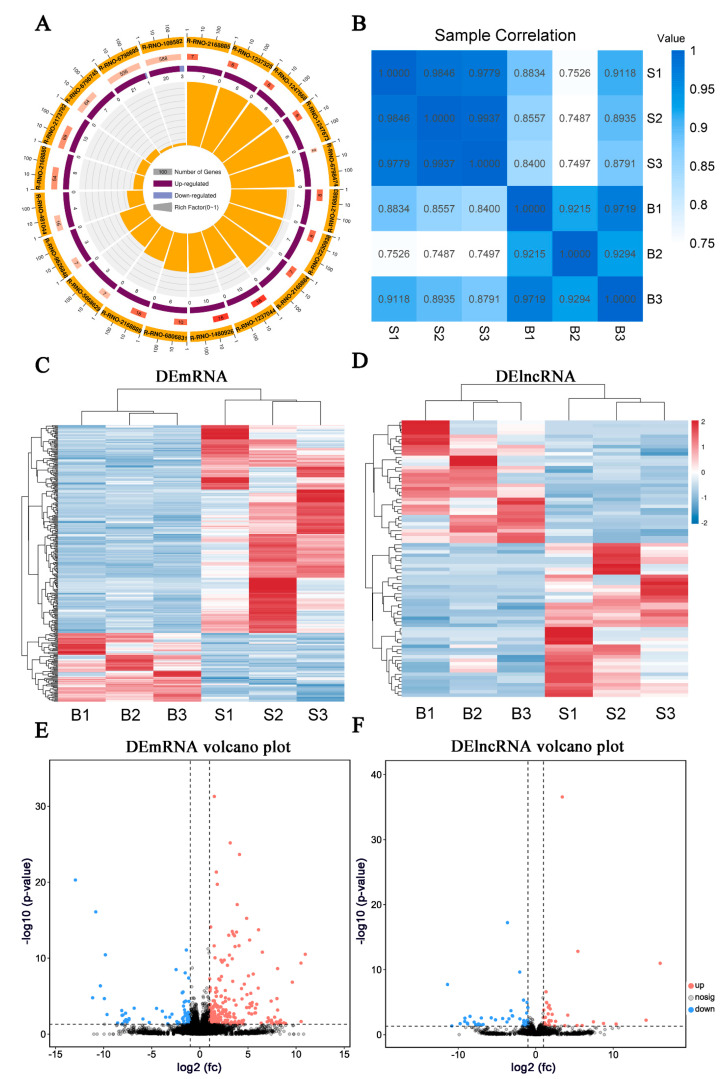
Significant changes in mRNA and lncRNA expression identified by high-throughput transcriptomic sequencing in rats with BCP–depression comorbidity. (**A**) Circos plot showing the overall distribution of the detected reads in the genome. S1, S2, S3: triplicate samples of the sham group; B1, B2, B3: triplicate samples of the BCP–depression group. (**B**) The correlation heatmap of gene expression was assessed using Pearson’s correlation coefficient for each sample between the two groups. (**C**) Hierarchical cluster analysis of DEmRNAs. The color (from blue to red) represents gene expression intensity (log10 FPKM) from low to high, indicating downregulation and upregulation, respectively. (**D**) Hierarchical cluster analysis of DElncRNAs. (**E**) Volcano plot of DEmRNAs, where vertical lines correspond to 2-fold changes in upregulation or downregulation; the horizontal line represents q = 0.05, and the *p*-value is adjusted by FDR; red points indicate mRNAs with upregulation and blue points indicate mRNAs with downregulation. (**F**) Volcano plot of DElncRNAs.

The expression patterns of mRNAs and lncRNAs in the amygdala of BCP–depression and sham rats were evaluated. The transcripts in each sample were distributed almost equally on all chromosomes, and the upregulated genes constituted the majority of the transcripts (Figure 3A). All transcript expression levels were calculated using Pearson’s correlation coefficient and a correlation heatmap of differences between all samples, showing a distinct difference between BCP–depression and sham rats but high intragroup congruence (Figure 3B).

A total of 27,095 mRNAs and lncRNAs were identified from the RNA-sequencing data. The selected criteria for DEGs were *p*-value < 0.05, FDR < 0.05, and fold change >2 or <−2. Finally, 330 DEmRNAs (248 with upregulated expression and 82 with downregulated expression) and 78 differentially expressed lncRNAs (DElncRNAs) (34 with upregulated expression and 44 with downregulated expression) were revealed by comparing the quantification of gene expression across the two groups. Moreover, there was a clear distinction shown in the hierarchical classification of mRNA and lncRNA expression between the BCP–depression and sham rats (Figure 3C,D). Volcano plots displayed the number and fold change in DEmRNAs and DElncRNAs (Figure 3E,F). Table 1 and Table 2 list the detailed data of the top 15 upregulated mRNAs and lncRNAs and the top 15 downregulated mRNAs and lncRNAs. These results indicate that the BCP–depression comorbidity caused by tumor implantation in the tibia changed the expression of genes in the contralateral amygdala.

### 3.3. Validation of DElncRNA and DEmRNA Changes per RT-qPCR Test

**Figure 4:**
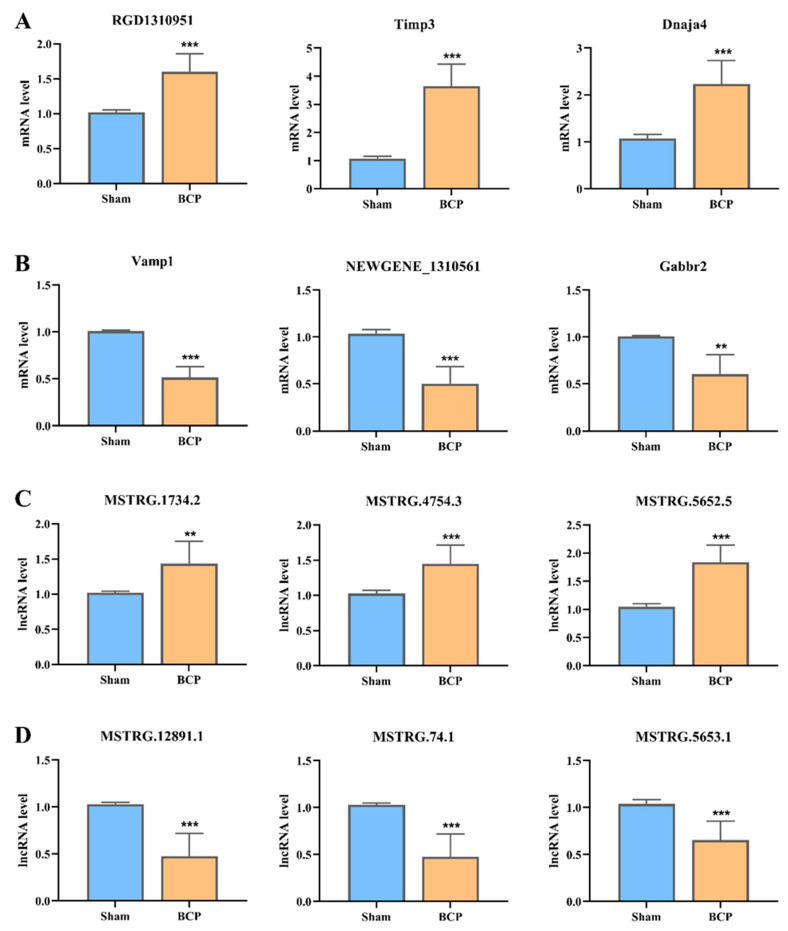
Independent validation of differential gene expression by RT-qPCR. (**A**) The expression levels of upregulated mRNAs. (**B**) The expression levels of downregulated mRNAs. (**C**) The expression levels of upregulated lncRNAs. (**D**) The expression levels of downregulated lncRNAs. BCP, bone cancer pain-depression comorbidity group. ** *p* < 0.01, *** *p* < 0.001 in comparison with sham rats, *n* = 7 per group.

To check the accuracy of the sequencing data, we selected 6 DEmRNAs (3 upregulated and 3 downregulated) and 6 DElncRNAs (3 upregulated and 3 downregulated) to conduct RT-qPCR to validate the reliability of our RNA-sequencing results on independent samples (*n* = 5 per group, Additional File 1). The quantitative PCR findings were in line with those of RNA-sequencing (Figure 4). RGD1310951, Timp3, and Dnaja4 were significantly upregulated, and Vamp1, NEWGENE_1310561, and Gabbr2 were significantly downregulated. Additionally, all three upregulated DElncRNAs, namely MSTRG.5652.5, MSTRG.4754.3, and MSTRG.1734.2, and three downregulated DElncRNAs, namely MSTRG.12891.1, MSTRG.74.1, and MSTRG.5653.1, showed consistent results with the RNA-sequencing findings.

### 3.4. Biological Functional Analysis of DEGs

**Figure 5:**
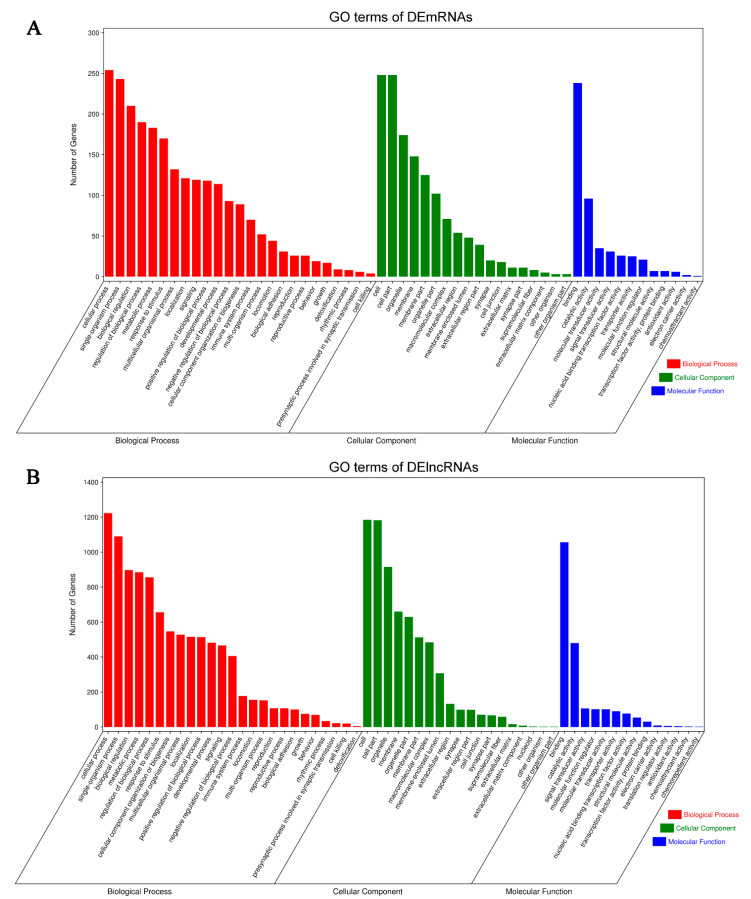
GO enrichment analysis of DEmRNAs and DElncRNAs. (**A**) Top 25 classes of GO enrichment terms for DEmRNAs according to the *p*-value, shared among the three ontologies: biological process (shown in red), cellular component (shown in green), and molecular function (shown in blue). (**B**) Top 25 classes of GO enrichment terms for a potential target of DElncRNAs.

The GO enrichment analysis of DEmRNAs indicated that the DEmRNAs were mainly enriched for the BP terms: cellular process, response to stimulus, and immune system process; the CC terms: cell part, membrane part, and macromolecular complex; and the MF terms: catalytic activity, nucleic acid binding transcription factor activity, and molecular transducer activity (Figure 5A). DElncRNAs were associated with the BP terms: single-organism process, metabolic process, and cellular component organization; the CC terms: cell part, organelle part, and membrane-enclosed lumen; and the MF terms: binding, catalytic activity, and signal transducer activity (Figure 5B).

**Figure 6:**
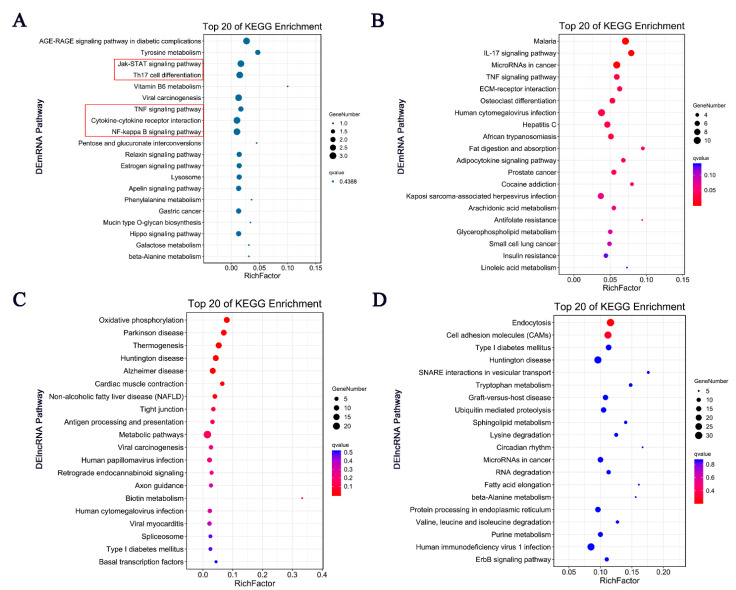
KEGG pathway enrichment analysis of DEmRNAs and DElncRNAs. (**A**) Top 20 pathways with upregulation in KEGG enrichment analysis. The size of the bubble indicates the number of enriched pathways, with colors indicating higher-level repeat classes. (**B**) Top 20 pathways with downregulation based on KEGG enrichment analysis. (**C**) KEGG enrichment of potential targets of DElncRNAs showing the top 20 pathways in trans. (**D**) KEGG enrichment of potential targets of DElncRNAs showing the top 20 pathways in cis. The red boxes indicate the differentially expressed pathways in KEGG that mainly represent inflammatory and immune responses.

Similarly, excluding the irrelevant pathways, KEGG analysis showed several representative enriched pathways of DEmRNAs, including the TNF signaling pathway, IL-17 signaling pathway, and microRNAs in cancer (Figure 6A,B). Additionally, potential target genes of DElncRNAs were concentrated in the processes of oxidative phosphorylation, endocytosis, antigen processing, and presentation (Figure 6C,D).

These results demonstrated the involvement of intracellular and extracellular pathways in inflammatory and immune processes as well as endocrine metabolism processes. The correlations between the GO enrichment and KEGG pathway analyses indicated that potential DEmRNA–DElncRNA interactions may occur in the development of BCP–depression comorbidity.

### 3.5. Western Blot Analyses of Proteins and Protein-Protein Interaction (PPI) Network Related to Immunity and Inflammation 

**Figure 7:**
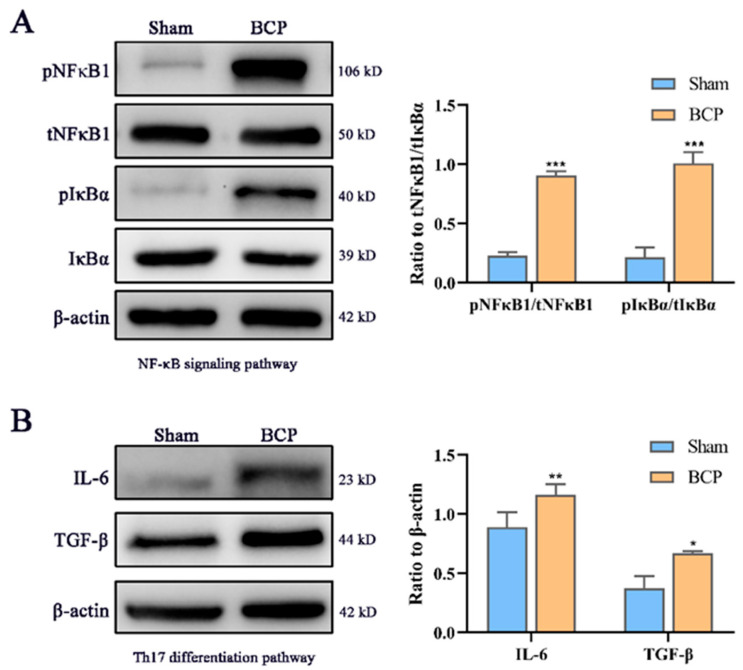
Western blot identification of expression changes in the NF-κB signaling pathway and Th17 differentiation pathway. (**A**) The protein NF-κB1 and IκBα from the NF-κB signaling pathway in BCP–depression rats were expressed at higher levels than those in sham rats. (**B**) The protein TGF-β and IL-6 (Th17 differentiation pathway) in BCP–depressed rats were higher than those in sham rats. pNF-κB1, phosphorylate NF-κB1; tNF-κB1, total NF-κB1; pIκBα, phosphorylate IκBα; tIκBα, total IκBα. BCP, bone cancer pain-depression comorbidity group; * *p* < 0.05, ** *p* < 0.01, and *** *p* < 0.001 compared with the sham group rats; *n* = 4 rats per group.

Four differentially expressed representatives, the NF-κB1 (p105/p50 subunit) and IκBα protein in NF-κB signaling and the TGF-β and IL-6 proteins in Th17 differentiation, were identified and selected to test the expression levels by Western blotting. The results showed that the protein expression levels of the phosphorylated NF-κB1 p105/p50 subunit and the phosphorylated IκBα were significantly increased compared with those of the sham group, while their total protein levels were minimally changed. TGF-β and IL-6 are necessary proteins during Th17 cell differentiation, and their expression levels were substantially increased (Figure 7).

**Figure 8:**
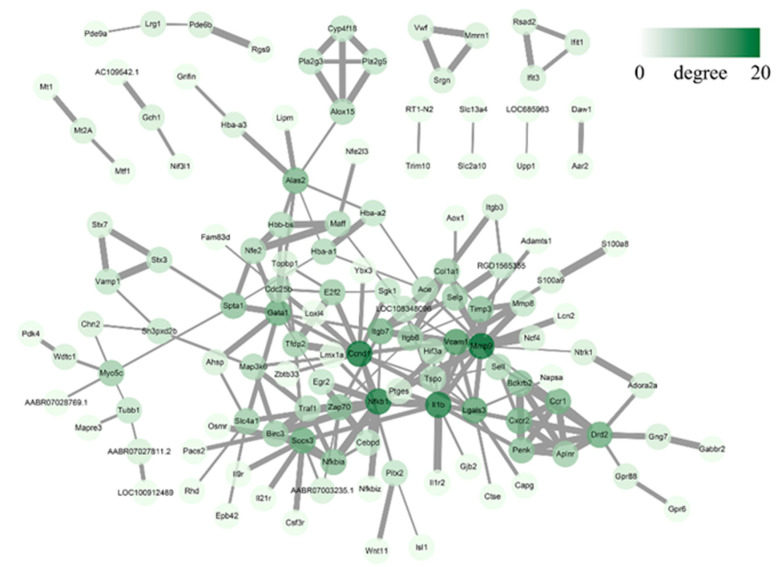
PPI network established with 200 DEmRNA-encoded proteins. The color scale of the node indicates the amount of connectivity with other nodes, and the thickness of the edge indicates the combined score of the interaction.

Moreover, the PPI network was constructed using the top 200 DEmRNAs corresponding proteins and associated interactions (Figure 8). A total of 129 nodes and 200 edges were mapped, and the five node proteins, namely matrix metalloprotein 9 (MMP-9), cyclin D1 (CCND1), NF-κB1, interleukin-1β (IL-1β), and suppressor of cytokine signaling 3 (SOCS3), which showed a close interaction with other node proteins, were chosen as hub proteins. Functional analysis of these interacting genes showed that they were predominantly concentrated in inflammatory and immunological processes, indicating that they were highly involved in the pathogenesis of the BCP–depression comorbidity.

## 4. Discussion

In this study, we observed both BCP and depressive behaviors after implantation of MRMT-1 carcinoma cells. As the central site of depression and pain, the amygdala integrates and modulates the nociception signal and brings forth extensive alteration on molecular, cellular, and functional levels in the BCP–depression comorbidity condition. Given that the biological mechanisms of most lncRNAs remain unknown, coupled with the limited number of studies about their roles in BCP–depression comorbidity, RNA sequencing was conducted to assess the overall view of gene expression changes in the current study, which detected 330 DEmRNAs (248 with upregulated expression and 82 with downregulated expression) and 78 DElncRNAs (34 with upregulated expression and 44 with downregulated expression).

Several lncRNAs have been functionally characterized in previous research on pain or depression, such as XIST, uc.48+, NEAT1, and NONRATT007487.2 in neuropathic pain [13,27] and DISC2, BACE1-AS, and BDNF-AS in depression [28,29,30]. Most of these lncRNAs are involved in the inflammatory response and oxidative stress. The present study first explored novel lncRNAs that changed their expression in a BCP–depression comorbidity model that showed similar results to those of our study. We found several DElncRNAs that regulate the inflammation axis and oxidative process, such as SLC25a52 [31], Zbtb5 [32], and SMG1 [33]. Some have been reported as participants in schizophrenia and neurodegenerative diseases, such as CACNA1I [34] and PNPLA8 [35]. However, the above-mentioned lncRNAs were not identified in the top 15 differentially expressed changes in the present study. The reasons for this are not clear, but it is likely due in part to differences in sample collection and the experimental animal model. For example, the highly differential model for SMG1 is sepsis, the model for SLC25a52 is secondary renal amyloidosis, and the model for Zbtb5 is non-small-cell lung cancer [31,32,33]. 

Mounting evidence highlights the involvement of inflammatory/immune responses and their relationships in depression and pain research [36,37,38]. Displaying high degrees of coherence with previous studies, the GO enrichment and KEGG analysis of potential target genes of DElncRNAs and DEmRNAs in our study were mainly involved in inflammatory and immune pathways, represented by Th17 cell differentiation and the NF-κB signaling pathway.

As common neural mediators exist between pain and depression, it is assumed that inflammation induces a complex network of immune-to-brain signaling that regulates behavioral production during illness [39]. Clinicopathologic studies indicate that the central nervous system (CNS) may not be an “immune-privileged” organ, especially in certain pathologic conditions [40,41]. When systemic inflammation is activated, the permeability of the blood–brain barrier (BBB) can be reinforced by specific transport mechanisms [42]. Cytokines, such as TNF-α and IL-1β, can directly interact with the neural environment and also target downstream pathways that may interfere with neural function [43].

Consequently, Th17 cells, one of the differentiated subtypes of CD4+ T cells, can infiltrate the spinal cord through BCP and cause an imbalance in the Th17/Treg cell ratio in the spinal cord [44]. Coincidentally, depressed patients showed an imbalance between Treg cells and Th17 cells following chronic exposure to stressors, and these responses may be associated with worsening depressive symptoms [45]. TGF-β and IL-6 efficiently induce the production of the lineage-specific transcription factors RORγt and IL-17 [46]; thus, we identified the protein expression of TGF-β and IL-6 to further validate the RNA-seq data, and the results showed that their expression was increased.

Similarly, as NF-κB is a key transcription factor that regulates the expression of inflammatory and immune response genes [47], the activation of the NF-κB pathway has been demonstrated to be associated with BCP [48]. Several studies have also confirmed that the NF-κB signaling pathway plays a key role in the development of depression [49,50], especially subunit NF-κB1 (p105/p50), an important regulator of in vivo NF-κB activity [51]. p105 can generate p50 by signaling-induced, ubiquitin-dependent limited proteolysis. p50 homodimers play an active anti-inflammatory role in canonical NF-κB activation pathways, whereas the p105 precursor has a variety of independent NF-κB functions [51,52]. IκBα acts as a key molecule of IκB kinase, the phosphorylation and degradation of which promote the activation of NF-κB [53]. Therefore, we determined the protein expression of NF-κB1 and IκBα, and the results revealed that the levels of phosphorylated NF-κB1 and phosphorylated IκBα were significantly increased compared with those of NF-κB and IκBα. Overall, we demonstrated variation in the trends of some factors and pathways that have been reported to have roles in previous studies. These findings can help us better understand the epigenetic processes of the BCP–depression comorbidity.

Using PPI network analyses, we identified five hub proteins: MMP-9, CCND1, NFκB1, IL-1β, and SOCS3. The results concerning these hub proteins are mostly in agreement with previously reported experimental studies, which found that they may play a pivotal role in the inflammatory effects of BCP–depression comorbidity. For instance, the activation of MMP-9 in the brain led to increased BBB permeability, which is the beginning of the invasion of peripheral immune cells into the CNS [54,55]. In addition, the inhibition of the NF-κB signaling pathway and its downstream inflammatory factor IL-1β suppressed oxidative stress and inflammation in the developmental process of bone cancer pain [56,57]. Moreover, SOCS3-induced suppression of the JAK2/STAT3 pathway attenuated neuropathic pain [58].

Through sequencing, we found that these lncRNAs were mainly enriched in the inflammatory and immunological pathways. However, the role of certain lncRNAs in BCP–depression comorbidity was not specifically discussed, which is a limitation of our study. Another limitation of our research that should be addressed in future work is the lack of mechanistic studies. In addition, it should be recognized that although we implemented rigorous quality control at each of the key milestones and the results have been confirmed by conventional reverse transcription-quantitative polymerase chain reaction, many factors have the potential to influence the quantification of RNA sequences. Consequently, a strict experimental check is essential to obtain precise conclusions.

## 5. Conclusions

In conclusion, this is the first study providing abnormal lncRNA and mRNA expression profiles in the amygdalae of rats with BCP–depression comorbidity. Our data indicate that the differentially expressed genes are predominantly enriched in the inflammatory and immunological pathways. Our study may provide novel insights and lay a theoretical foundation for future research on the pathogenesis of BCP and depression comorbidity.

## Figures and Tables

**Table 1:** Detailed data of the top 15 upregulated mRNAs and the top 15 downregulated mRNAs.

Gene Name	Description	log2FC	*p*-Value
Upregulation			
RGD1310951	similar to RIKEN cDNA	>10	3.15 × 10 ^−11^
Timp3	TIMP metallopeptidase inhibitor 3	>10	4.60 × 10^−10^
Dnaja4	DnaJ heat shock protein family (Hsp40) member A4	9.60	1.46 × 10^−7^
S100a8	S100 calcium binding protein A8	6.09	1.82 × 10^−14^
Cxcr2	C-X-C motif chemokine receptor 2	6.07	9.09 × 10^−7^
Cd177	CD177 molecule	5.91	1.14 × 10^−4^
Rhag	Rh-associated glycoprotein	5.77	3.99 × 10^−4^
Gata1	GATA binding protein 1	8.08	6.78 × 10^−4^
Mmp8	matrix metallopeptidase 8	5.54	1.21 × 10^−8^
S100a9	S100 calcium binding protein A9	8.07	2.41 × 10^−9^
Arntl	aryl hydrocarbon receptor nuclear translocator-like	8.00	2.11 × 10^−5^
Ngp	neutrophilic granule protein	5.45	7.08 × 10^−5^
Mcemp1	mast cell-expressed membrane protein 1	5.22	9.90 × 10^−5^
Nlrp12	NLR family, pyrin domain containing 12	5.13	3.1 × 10^−^^4^
Nfkb1	nuclear factor kappa B subunit 1	7.09	7.068 × 10^−^^3^
Downregulation			
Vamp1	vesicle-associated membrane protein 1	<−10	5.20 × 10^−21^
NEWGENE_1310561	programmed cell death 5	<−10	1.63 × 10^−5^
Gabbr2	gamma-aminobutyric acid type B receptor subunit 2	<−10	8.08 × 10^−17^
Lgals3	galectin 3	<−10	4.33 × 10^−7^
Tmem8b	transmembrane protein 8B	−9.92	2.12 × 10^−5^
Kif26a	kinesin family member 26A	−9.81	3.65 × 10^−11^
Paqr8	progestin and adipoQ receptor family member 8	−8.11	7.99 × 10^−4^
Dcun1d2	defective in cullin neddylation 1 domain containing 2	−8.08	3.428 × 10^−^^3^
Sertad2	SERTA domain containing 2	−7.98	3.115 × 10^−^^3^
Ccdc92	coiled-coil domain containing 92	−7.71	1.756 × 10^−3^
Vcam1	vascular cell adhesion molecule 1	−1.73	6.764 × 10^−^^3^
Cpxm2	carboxypeptidase X	−2.62	5.99 × 10^−5^
Zfp280c	zinc finger protein 280C	−7.35	9.41 × 10^−3^
Capn5	calpain 5	−6.82	3.8 × 10^−4^
LOC100910708	aldose reductase-related protein 1-like	−4.50	4.18 × 10^−4^

**Table 2:** Detailed data of the top 15 upregulated lncRNAs and the top 15 downregulated lncRNAs.

LncRNA ID	Chromosomal Locus	log2FC	*p*-Value
Upregulation			
MSTRG.5652.5	14:46638968-46643403	>10	5.12 × 10^−9^
MSTRG.4754.3	12:44294988-44296879	5.44	1.01 × 10^−10^
MSTRG.16920.1	9:1433268-1436339	1.88	1.13 × 10^−4^
MSTRG.1734.2	1:215913711-215968992	3.43	5.45 × 10^−34^
MSTRG.7026.1	16:66316521-66324304	1.75	3.15 × 10^−5^
MSTRG.13760.6	5:156317010-156325525	1.64	6.11 × 10^−5^
MSTRG.13760.7	5:156317010-156325525	1.64	6.11 × 10^−5^
ENSRNOT00000092949	1:221158098-221158792	>10	6.117 × 10^−3^
MSTRG.14298.2	6:69986056-69996290	2.57	8.838 × 10^−3^
MSTRG.5.2	1:1671268-1675416	1.99	7.356 × 10^−3^
ENSRNOT00000079224	16:60008769-60018283	1.74	6.637 × 10^−3^
ENSRNOT00000088948	16:59997072-60000092	8.75	1.8916 × 10^−2^
MSTRG.1478.10	1:194865923-194974619	7.45	1.088 × 10^−2^
MSTRG.5207.3	13:84531041-84536203	10.38	2.3133 × 10^−2^
MSTRG.4300.2	12:3340366-3341210	1.42	1.24 × 10^−5^
Downregulation			
MSTRG.74.1	1:11962604-11967166	−3.66	6.00 × 10^−18^
MSTRG.14169.1	6:30629736-30630859	−2.06	2.34 × 10^−10^
MSTRG.12891.1	5:23347609-23377614	<−10	1.91 × 10^−8^
MSTRG.5653.1	14:46644977-46686204	−3.20	2.05 × 10^−4^
MSTRG.5652.3	14:46635183-46683415	−3.03	1.256 × 10^−3^
MSTRG.14261.1	6:52444440-52459777	−1.57	4.80 × 10^−6^
MSTRG.10204.1	20:7065377-7156807	−8.48	1.507 × 10^−3^
MSTRG.1810.2	1:220139299-220144318	−9.14	3.027 × 10^−3^
MSTRG.16973.1	9:10509195-10537693	−1.51	8.48 × 10^−3^
MSTRG.13242.1	5:91131894-91135016	−1.53	6.741 × 10^−3^
MSTRG.12287.1	4:112115082-112140886	−2.04	3.733 × 10^−3^
ENSRNOT00000092903	5:60538928-60540538	−2.98	1.4 × 10^−3^
MSTRG.17595.5	9:113964702-113968568	−3.45	3.773 × 10^−3^
ENSRNOT00000092608	7:121511097-121619283	−5.74	2.635 × 10^−3^
ENSRNOT00000077174	4:16946938-16953847	−7.02	2.78 × 10^−3^

## Data Availability

Data are available from the corresponding author upon specific request.

## Supplementary Materials

The following are available online at https://www.mdpi.com/article/10.3390/life11080834/s1, Table S1: Sequences of primers for RT-qPCR.
