# Supplementary material for: Transcriptomic Analysis of Long Noncoding RNA and mRNA Expression Profiles in the Amygdala of Rats with Bone Cancer Pain-Depression Comorbidity
Source: Life (Basel). 2021 Aug 14;11(8):834. doi: 10.3390/life11080834 (PMC8400935; doi:10.3390/life11080834)
Supplement: Supplementary file 1 [file life-11-00834-s001.zip › life-1306062-supplementary.pdf]

life

MDPI

Article

Transcriptomic Analysis of Long Noncoding RNA and mRNA Expression Profiles in the Amygdala of Rats with Bone Cancer Pain–Depression Comorbidity

Shuyan Wu [1,2,†], Xiaohui Chen [1,2,†], Fengyi Huang [1,2,†], Mingxue Lin [1,2], Pinzhong Chen [1,2], Haiyang Wan [1,2], Fei Gao [1,2], Ting Zheng [1,2] and Xiaochun Zheng [1,2,3,*]

1 Department of Anesthesiology, Shengli Clinical Medical College of Fujian Medical University, Fuzhou 350001, China; 2191003810@fjmu.edu.cn (S.W.); chenxh@fjmu.edu.cn (X.C.); huangfy@fjmu.edu.cn (F.H.); ccdjzzi@fjmu.edu.cn (M.L.); chenpinzhong@fjmu.edu.cn (P.C.); 2191003809@fjmu.edu.cn (H.W.); gaofei@fjmu.edu.cn (F.G.); zhengting@fjsl.com.cn (T.Z.)

2 Department of Anesthesiology, Fujian Provincial Hospital, Fuzhou 350001, China

3 Fujian Provincial Institute of Emergency Medicine, Fujian Provincial Key Laboratory of Emergency Medicine, Fuzhou 350001, China

* Correspondence: zhengxiaochun@fjsl.com.cn

† Authors contributed equally to this work.

Citation: Editor, A.; Liu, F.-C. Transcriptomic Analysis of Long Noncoding RNA and mRNA Expression Profiles in the Amygdala of Rats with Bone Cancer Pain–Depression Comorbidity. *Life* **2021**, *11*, 834. https://doi.org/10.3390/life11080834

Academic Editors: Huang-Ping Yu and Fu-Chao Liu

Received: 3 July 2021
Accepted: 11 August 2021
Published: 14 August 2021

Publisher's Note: MDPI stays neutral with regard to jurisdictional claims in published maps and institutional affiliations.

Copyright: © 2021 by the authors. Licensee MDPI, Basel, Switzerland. This article is an open access article distributed under the terms and conditions of the Creative Commons Attribution (CC BY) license (http://creativecommons.org/licenses/by/4.0/).

Supplementary Materials:

Table S1. Sequences of Primers in RT-qPCR.

Gene Name	Primer Sequences (5′-3′)
RGD1310951	Forward: CCTCGTCTGGCTCAACAATAC<br>Reverse: AGTCTGTCCCACCTCTCTTG
Timp3	Forward: GCTGTGCAACTTTGTGGAGAG<br>Reverse: ACCCGAAATTGGAGAGCATGT
Dnaja4	Forward: GATCTCAGAGCGGCCTAACC<br>Reverse: AGCTCCAGTTCTGATTGCCG
Vamp1	Forward: GGTCCTCCTCCCAATACGAC<br>Reverse: CAGCTCGGTCATCCAACTCT
NEWGENE_1310561	Forward: GCCTGAGAAAACGAAAGCAGT<br>Reverse: TCTGTTGGCTGACTTTCTCG
Gabbr2	Forward: ACTACACAGACCACACGCTG<br>Reverse: TGTATTCGCCGACCTTCACC
MSTRG.12891.1	Forward: TCCTGAGTGCGAGTTAGTGT<br>Reverse: GCTTTGCTTGGTGTTGAGAATG
MSTRG.74.1	Forward: AGGAGTTGAGTGTGCGGTTA<br>Reverse: AGGAGGAGCCTTGACTTGAG
MSTRG.5653.1	Forward: CTGTGTGAGTGTTCCTCCTTCT<br>Reverse: CGTCAAGGGAAGAGCGAGAA
MSTRG.1734.2	Forward:CCAGTGTAATCTTGCCAGAAGTT<br>Reverse:GGAATGTAGGGATGGGAATGAAG
MSTRG.4754.3	Forward: GACATATCTGCGTGGTTCTGG<br>Reverse: GGCTCCTGACTACAATCTCCTA
MSTRG.5652.5	Forward: CGCTCCTTACCTGGTTGATC<br>Reverse: CGACCAAAGGAACCATAACTGA
GAPDH	Forward: ACGGCAAGTTCAACGGCACAG<br>Reverse:GAAGACGCCAGTAGACTCCACGAC
